# Protein Tyrosine Phosphatase 1B Deficiency Ameliorates Murine Experimental Colitis via the Expansion of Myeloid-Derived Suppressor Cells

**DOI:** 10.1371/journal.pone.0070828

**Published:** 2013-08-09

**Authors:** Jing Zhang, Bing Wang, Wen Zhang, Yao Wei, Zhen Bian, Chen-Yu Zhang, Limin Li, Ke Zen

**Affiliations:** 1 Jiangsu Engineering Research Center for MicroRNA Biology and Biotechnology, State Key Laboratory of Pharmaceutical Biotechnology, Nanjing University School of Life Sciences, Nanjing, Jiangsu, China; 2 People’s Liberation Army 404 Hospital, Weihai, Shandong, China; French National Centre for Scientific Research, France

## Abstract

Protein tyrosine phosphatase 1B (PTP1B) is a key molecule in modulating low-degree inflammatory conditions such as diabetes. The role of PTP1B in other chronic inflammations, however, remains unknown. Here, we report that PTP1B deficiency ameliorates Dextran Sulfate Sodium (DSS)-induced murine experimental colitis via expanding CD11b^+^Gr-1^+^ myeloid-derived suppressor cells (MDSCs). Employing DSS-induced murine experimental colitis as inflammatory animal model, we found that, compared with wild-type littermates, PTP1B-null mice demonstrated greater resistance to DSS-induced colitis, as reflected by slower weight-loss, greater survival rates and decreased PMN and macrophage infiltration into the colon. The evidence collectively also demonstrated that the resistance of PTP1B-null mice to DSS-induced colitis is based on the expansion of MDSCs. First, PTP1B-null mice exhibited a greater frequency of MDSCs in the bone marrow (BM), peripheral blood and spleen when compared with wild-type littermates. Second, PTP1B levels in BM leukocytes were significantly decreased after cells were induced into MDSCs by IL-6 and GM-CSF, and the MDSC induction occurred more rapidly in PTP1B-null mice than in wild-type littermates, suggesting PTP1B as a negative regulator of MDSCs. Third, the adoptive transfer of MDSCs into mice with DSS-colitis significantly attenuated colitis, which accompanies with a decreased serum IL-17 level. Finally, PTP1B deficiency increased the frequency of MDSCs from BM cells likely through enhancing the activities of signal transducer and activator of transcription 3 (STAT3) and Janus kinase 2 (JAK2). In conclusion, our study provides the first evidences that PTP1B deficiency ameliorates murine experimental colitis via expanding MDSCs.

## Introduction

PTP1B serves as a major negative regulator of insulin and leptin sensitivities by dephosphorylating the insulin receptor and the leptin receptor-associated JAK2 [1]. Widely expressed in many cell types and tissues, including skeletal muscle, liver, adipose tissue, brain, and immune cells [1], PTP1B can also dephosphorylate more distal components of the insulin and leptin signaling pathways, such as insulin receptor substrate 1 [2], [3]. Recent studies have also demonstrated that PTP1B is expressed in immune cells [4], [5], which strongly suggests a critical role for PTP1B in modulating leukocyte inflammatory responses. Because obesity and diabetes are regarded as inflammatory states characterized by the elevation of the pro-inflammatory cytokines Tumor necrosis factor α (TNFα), interleukin-1, and interleukin-6 in adipose tissue or sera [6], PTP1B may serve as an inflammatory target during obesity-associated inflammation [7]–[9]. In humans and rodents, obesity-associated inflammation is characterized by a significant infiltration of macrophages into adipose tissue, where the macrophages serve as potent sources of pro-inflammatory cytokines [7], [9]. Under pathophysiologic conditions such as insulin resistance, obesity, and diabetes, PTP1B is often over-expressed in the liver, adipose tissue, muscle, and the nucleus of hypothalamus, suggesting that PTP1B may increase in response to certain inflammatory factors. The excellent work conducted by Zabolotny et al. [8] further demonstrated that TNFα treatment alone is sufficient to increase PTP1B mRNA and protein levels in cultured cells and the insulin- and leptin-targeted tissues of mice. Although these studies suggested that the inhibition of PTP1B activity may attenuate obesity-associated inflammation, the ability of PTP1B deficiency to protect against other chronic inflammatory conditions, such as colitis, remained unknown.

Myeloid-derived suppressor cells (MDSCs) represent a heterogeneous population of immature myeloid cells, including myeloid progenitors, macrophage precursors and granulocytes and dendritic cells, which are characterized by a strong ability to suppress various T cell functions [10], [11]. In mice, MDSCs are characterized by the co-expression of Gr-1 and CD11b [11]. The inhibitory properties of MDSCs are proposed to be mediated by various mechanisms, including increased levels of reactive oxygen species (ROS) and the expression of inducible NOS and ARG, which both participate in the metabolism of L-arginine [11], [12]. In murine studies, a small number of CD11b^+^Gr-1^+^ cells (4%) can be detected in the blood and the spleens of naive wild-type mice. However, the frequency of MDSCs increases dramatically under pathological conditions such as tumor growth and graft-versus-host disease [12]. The expansion of MDSCs and its protective role in suppressing body inflammation and autoimmunity has also been observed in various pathophysiological conditions [13]–[15]. Recently, Singh et al. [16] demonstrated that resveratrol induces the accumulation of CD11b^+^Gr-1^+^ MDSCs, which reduces the number of CXCR3^+^ T cells and ameliorates chronic colitis in IL-10-deficient mice. Furthermore, studies conducted by Haile et al. [17] identified MDSCs as a critical component in a novel immune-regulatory pathway in inflammatory bowel disease (IBD). By co-transferring MDSCs with HA-specific CD8^+^ T cells into naive VILLIN-HA mice, Haile et al. [17] reported that enterocolitis induced by antigen-specific T cells was ameliorated. Given that both MDSCs and PTP1B deficiency can attenuate obesity-associated inflammation, it is logical to speculate that the loss of PTP1B may contribute to the expansion of MDSCs. However, reports describing the potential linkage between PTP1B deficiency and the expansion of MDSCs are currently lacking.

In the present study, we characterized the role of PTP1B in murine experimental colitis. By comparing DSS-induced colitis in PTP1B-null mice and their wild-type littermates, we determined that PTP1B-deficient mice are more resistant to DSS-induced colitis than their wild-type littermates and that this resistance likely occurs via the expansion of MDSCs, which is promoted by deficient PTP1B. The protective effect of MDSCs on experimental colitis was analyzed after the adoptive transfer of MDSCs into mice or following treatment with glucocorticoids, which also increases the frequency of mouse MDSCs. The mechanism underlying MDSC expansion in the absence of PTP1B was further explored in the study.

## Materials and Methods

### Ethics Statement

All animal experimental procedures were carried out in accordance with the National Institutes of Health Guide for the Care and Use of Laboratory Animals and were approved by the Animal Care Committee of Nanjing University (Nanjing, China).

### Reagents and Antibodies

The murine cytokines IL-6, GM-CSF and TNFα were purchased from Pepro Tech (Rocky Hill, NJ, USA). The anti-PTP1B antibody was purchased from Abcam (Cambridge, MA). The anti-JAK2, anti-p-JAK2 and anti-GAPDH antibodies, as well as the PTP1B inhibitor, were purchased from Santa Cruz Biotechnology (Santa Cruz, CA). PE-conjugated anti-mouse CD11b and PerCP/Cy5.5-conjugated anti-mouse Gr-1 were purchased from BioLegend (San Diego, CA). FITC-conjugated rat anti-mouse CD4 and CD8a, APC-conjugated anti-mouse Ly6G and Alexa Fluor 488-conjugated anti-mouse Ly-6C antibodies were purchased from BD Biosciences (San Jose, CA). The anti-mouse F4/80 antibody and IL-17A were purchased from eBioscence (San Diego, CA). The IL-6, IL-12 p40/70, IL-10, and GM-CSF ELISA kits were purchased from R&D Systems (Minneapolis, MN).

### Mice and the DSS Colitis Mouse Model

Male C57BL/6J mice (6–8 weeks old, weighing 18–22 g) were purchased from the Model Animal Research Center of Nanjing University (Nanjing, China) and maintained in a pathogen-free animal facility on a 12 h light/dark cycle. PTP1B-deficient mice with C57 background were obtained from Dr. Benjamin G. Neel (Harvard Medical School, Boston, Massachusetts). Dextran sulfate sodium (DSS)-induced mouse colitis was established as previous described with monor modification [18]–[20]. Briefly, 3.5% DSS (MW 36 000–50 000; MP Biomedicals) was dissolved in filter-purified and sterilized drinking water and administered to the mice daily. Control mice were given normal drinking water. Mice were monitored for weight loss and pathological features (rectal bleeding and diarrhoea). The presence of diarrhoea, rectal bleeding, and weight loss were graded separately on a 0–4 scale, and the sum of the three values constitutes the disease activity index (DAI) [18]. Mice were euthanized by CO_2_ followed by cervical dislocation. After removing caecum, the remainder of the colon was divided into proximal and distal halves. Tissue was fixed in 10% buffered formalin, paraffin-embedded, sectioned, and stained with haematoxylin and eosin.

### Mouse Bone Marrow Culture

Bone marrow (BM) was harvested from the femurs of C57BL/6 mice. Red blood cells (RBCs) were lysed using ammonium chloride and EDTA. To obtain BM-derived MDSCs, cells were cultured in RPMI 1640 medium supplemented with 2 mM L-glutamine, 10 mM HEPES, 20 µM 2-mercaptoethanol, 150 U/ml streptomycin, 200 U/ml penicillin, and 10% fetal bovine serum and then stimulated with combinations of GM-CSF and IL-6 (40 ng/ml each) [21]. Cells were maintained at 37°C in 5% CO_2_-humidified atmosphere for 4 days. TNFα was used at concentrations of 0, 0.1, 1.0 and 10 nM. The PTP1B inhibitor was used at a concentration of 20 µM.

### Blood and Spleen Cell Preparation

Peripheral blood samples were collected at 1.5 ml tube containing 3.8% sodium citrate anticoagulant. Peripheral blood mononuclear cells were isolated from fresh blood by Ficoll gradient separation [22]. For isolation of spleen cells, spleens were collected in sterile HBSS without Ca^2+^ and Mg^2+^, grinded and filtered. After depleting erythrocytes, purified splenic cells were collected for further flow analysis.

### Flow Cytometry Analysis of MDSCs and Th-17 Cells

After washing with Hank’s buffer devoid of Ca^2+^ and Mg^2+^ (HBSS), 5×10^5^ cells from BM, spleen, and peripheral blood were blocked in 100 µl of 1% BSA at 4°C for 30 minutes. For the analysis of MDSCs, leucocytes were surface labeled with fluorescently conjugated antibodies against CD11b, Gr-1, Ly-6G, or Ly-6C (eBioscience). For the analysis of Th17 cells, PBMCs were suspended at a density of 2×10^6^ cells/mL in complete culture medium. Cells were stimulated for 5 h using 50 ng/mL of phorbol myristate acetate (Sigma-Aldrich) and 1 g/mL ionomycin (Sigma-Aldrich) in the presence of 5 g/mL Brefeldin A (Sigma-Aldrich) at 37°C. The cells were then washed in phosphate-buffered saline (PBS) and surface-labeled with anti-CD4-FITC (eBioscience). Following surface staining, the cells were fixed and permeabilized using IntraPrep Permeabilization Reagent (Beckman Coulter Inc.) and then stained with anti-IL-17A-PE (eBioscience) [23]. Labeled cells were washed and analyzed with a FACSCalibur flow cytometer (Becton-Dickinson) using the CellQuest software (Becton-Dickinson). In each case, staining was compared with that of the appropriately labeled isotype control antibody.

### Adoptive Transfer of BM-MDSCs

5×10^6^ MDSCs generated from mouse bone marrow (BM-MDSCs) by stimulation with IL-6 and GM-CSF were washed, collected, and then injected intravenously into each mouse on day 2 post-DSS treatment. After that, mice were injected intravenously with the same numbers of BM-MDSCs every 4 days.

### Immunohistochemistry and Myeloperoxidase (MPO) Assay

To detect tissue damage and macrophage infiltration, 5 µm sections of formalin-fixed, paraffin-embedded colon were de-paraffinized and rehydrated. Sections were stained with H&E or labeled using an anti-F4/80 antibody. To detect PMN infiltration, the MPO activity in homogenates of the colon was determined by the method of Zhao et al. [24]. Briefly, equal weights of the colon from each group were suspended in 50 mM phosphate buffer containing 0.5% hexadecyltrimethylammonium bromide (pH 6.0). After sonication (3×1 min, at 4°C), homogenates were centrifuged and the supernatants were stored at −80°C. The samples were incubated with a substrate of odianisidine hydrochloride and the reaction was carried out in a 96-well plate by adding to 50 mM phosphate buffer, substrate solution (containing 20 mg/mL odianisidine hydrochloride) and H_2_O_2_ (20 mM). The samples were added to each well to start the reaction. The reaction was stopped by adding sodium azide (30%) and the plates were read for the assay at light absorbance of 460 nm. MPO activity was determined by the curve obtained from the standard MPO.

### MDSC Sorting and Suppression of T cell Proliferation Assay

To obtain high purity MDSCs from the spleen, a cell isolation kit (Miltenyi Biotechnology, catalog no.: 130094538) was used according to manufacturer’s instructions. For the T cell inhibition assay, splenocytes were first separated using lymphocyte separation medium. Spleen CD4^+^ T cells were then isolated via negative selection using the CD4 isolation kit (Miltenyi Biotechnology). CD4^+^ T-cells were labeled with CFSE according to manufacturer’s instructions (Invitrogen). CFSE-labeled CD4^+^ T cells were then stimulated with anti-CD3 and anti-CD28, and T cells were co-cultured at a 2∶1 ratio with BM-MDSCs in 96-well ﬂat bottom plates. On day 4, cells were analyzed using flow cytometry.

### Measurement of Cytokines and Western Blot Analysis

Whole blood was collected in the absence of an anticoagulant, and the serum was isolated by centrifugation. Several cytokines, including IL-6, IL-12, p40/70, IL-17A, IL-10, and GM-CSF, were quantified in the serum samples using an ELISA kit (R&D Systems) according to the manufacturer’s instruction. The absorbance was measured with a microplate reader (Bio-Rad) using wavelength correction (A450 nm). BM cells were lysed in lysis buffer containing 100 mM Tris (pH 7.5), 150 mM NaCl, 1% Triton X-100, a protease inhibitor cocktail and PMSF. Sodium vanadate and sodium fluoride were included in the lysis buffer when extracted proteins were analyzed for changes in protein phosphorylation. Western blot analysis was conducted using antibodies specific for PTP1B, STAT3, p-STAT3, JAK2 or p-JAK2. The antigens were visualized using the ECL plus detection system (Amersham Pharmacia Biotech). Normalization was performed by blotting the same samples with an anti-GAPDH antibody.

### Statistical Analysis

All flow cytometry and Western blot data are representative of at least three independent experiments. The data are presented as mean ± SD from at least three independent experiments. Differences are considered statistically significant if *p*<0.05 by Student’s *t* test.

## Results

### The Loss of PTP1B Attenuates DSS-induced Murine Experimental Colitis

**Figure 1:**
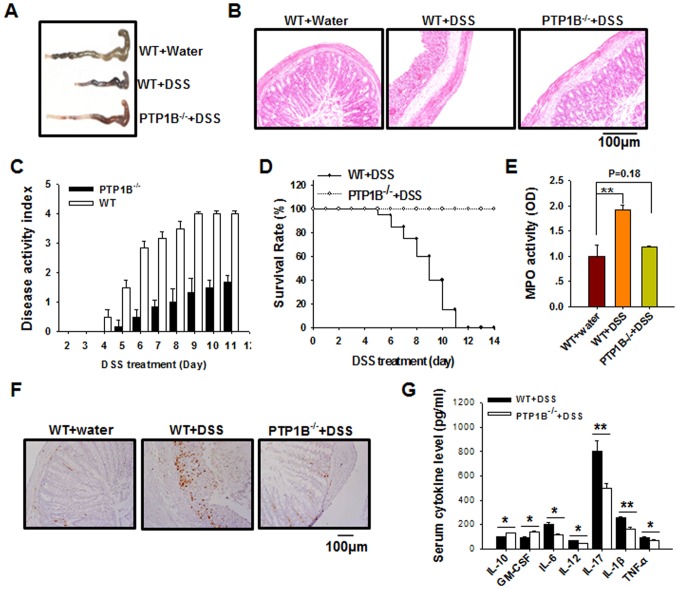
PTP1B^−/−^ mice are resistant to DSS-induced colitis. A) The colon tissue of WT and PTP1B^−/−^ mice that received either control water or water containing 2.5% DSS for 2 weeks. B) Representative H&E-stained mouse distal colonic sections on day 8 after treatment. C) Disease activity index after the beginning DSS treatment. D) The survival rate of mice during the course of DSS treatment. E-F) The relative activity of MPO (E) and the labeling with anti-F4/80 antibody (F) in colon from mice treated with or without DSS (on day 8). G) Serum cytokine concentrations in WT and PTP1B^−/−^ mice after 8 days of DSS treatment. Data are representative of 3 replicated experiments that produced similar results (n = 6 per group per condition). *, *p*<0.05, **, *p*<0.01.

PTP1B^−/−^ mice and their WT littermates received 2.5% DSS in their drinking water to induce colitis. Multiple observations collectively indicated that PTP1B^−/−^ mice were considerably more resistant to DSS-induced colitis than WT littermates. In WT mice, DSS treatment caused severe rectal bleeding, diarrhea, and weight loss toward the end of the treatment. However, these clinical indicators were not observed in PTP1B^−/−^ mice. As shown in Figure 1A, shortening of the colon, which is a macroscopic indication of colitis, was observed in DSS-treated WT mice but not DSS-treated PTP1B^−/−^ mice. Histological examination of colonic sections revealed complete disruption of the colonic architecture in WT mice, whereas PTP1B^−/−^ mice retained intact colonic architecture (Figure 1B). Consistent with these results, PTP1B^−/−^ mice generally exhibited a lower DAI (Figure 1C) and a higher survival rate (Figure 1D) than WT mice during the course of DSS treatment. Additionally, DSS treatment stimulated an increase in the colonic activity of myeloperoxidase (MPO), an indicator of neutrophil infiltration, in WT mice but not in PTP1B^−/−^ mice (Figure 1E). In agreement with this result, significantly less macrophage infiltration was observed in the colon tissue from PTP1B^−/−^ mice compared with the colon tissue from WT mice (Figure 1F). Most importantly, as shown in Figure 1G, the serum levels of pro-inflammatory cytokines, including IL-6, IL-12, IL-1β, TNFα, and IL-17, were markedly lower in PTP1B^−/−^ mice than in WT mice following DSS treatment. In contrast, the serum levels of the anti-inflammatory cytokine IL-10 and GM-CSF were higher in the DSS-treated PTP1B^−/−^ mice than in the DSS-treated WT mice.

### PTP1B Deficiency Increases the Frequency of Functional MDSCs in Mice

**Figure 2:**
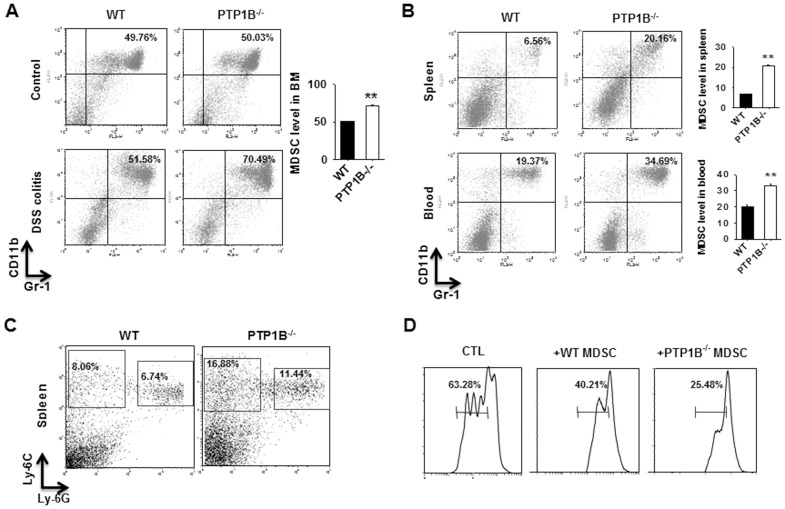
Increased functional MDSCs in PTP1B^−/−^ mice. A) The frequency of CD11b^+^Gr-1^+^ MDSCs in bone marrow of WT and PTP1B^−/−^ mice after DSS treatment for 8 days. B) The frequency of CD11b^+^Gr-1^+^ MDSCs in the spleens and blood of WT and PTP1B^−/−^ mice after DSS treatment for 8 days. C) The increased MDSCs in the spleen of PTP1B^−/−^ mice are both monocytic and neutrophil origin. D) The induced MDSCs in both PTP1B^−/−^ mice and WT mice inhibit T cell proliferation. Data are representative of 3 replicated experiments with similar results (n = 5 per group per condition). Compared with WT-DSS, *, *p*<0.05, **, *p*<0.01.

MDSCs were recently shown to play a critical role in modulating various types of inflammatory responses [10], [11], [17]. To determine whether MDSCs participate in the resistance to DSS-induced colitis in PTP1B^−/−^ mice, we quantified the frequency of MDSCs in mouse BM, spleen and peripheral blood using antibodies that recognized Gr-1 and CD11b, two markers of MDSCs [11]. As shown in Figure 2A, the basal frequency of MDSCs in the BM of PTP1B^−/−^ mice and WT littermates was almost the same. However, the population of MDSCs in the BM of both WT and PTP1B^−/−^ mice increased as DSS-induced colitis developed. Compared with WT mice, the frequency of MDSCs in the BM of PTP1B^−/−^ mice was significantly higher. Significantly higher frequency of MDSCs in PTP1B^−/−^ mice than in WT mice was also observed in mouse spleen and peripheral blood (Figure 2B). Additionally, labeling MDSCs with anti-Ly-6C and anti-Ly-6G antibodies further indicated that mouse MDSCs induced by DSS-colitis were derived from both monocytic and neutrophil origin (Figure 2C). An *in vitro* ConA-induced T cell proliferation assay confirmed that MDSCs in both WT and PTP1B^−/−^ mice treated with DSS were able to suppress the proliferation of T cells (Figure 2D). In support of this, we also found that the levels of arginase 1 (Arg1) and nitric oxide synthase 2 (NOS2) in BM-derived MDSCs were significantly higher than those in BM cells (data not shown).

**Figure 3:**
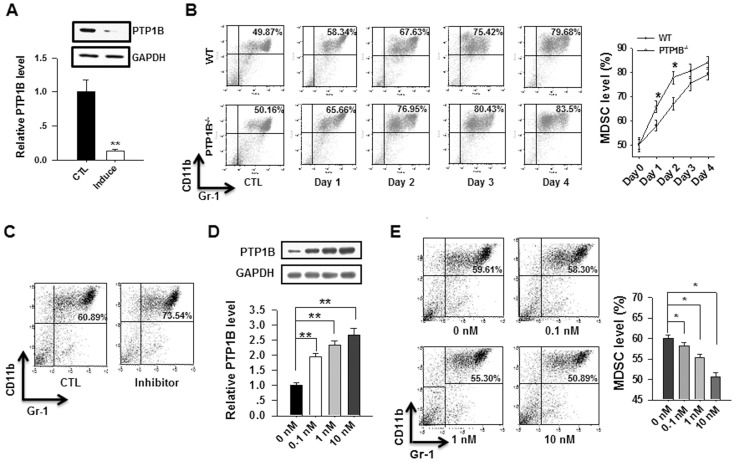
PTP1B is a negative regulator of MDSC induction. A) PTP1B protein expression in BM cells decreases upon MDSC induction by GM-CSF and IL-6 (40 ng/ml each). B) PTP1B^−/−^ BM cells cultured in the presence of GM-CSF and IL-6 give rise to MDSC more rapidly than WT BM cells. C) An inhibitor of PTP1B activity facilitates the induction of MDSC from WT mouse BM cells. D–E) TNFα increases PTP1B expression in BM cells (D) and inhibits the cytokine-induced conversion of MDSCs from BM cells. Data are representative of 3 replicated experiments with similar results (n = 5 per group per condition). Compared with WT, *, *p*<0.05, **, *p*<0.01.

Two different experiments were performed to further examine whether PTP1B deficiency promotes the generation of MDSCs. First, we monitored the levels of PTP1B in MDSCs generated from WT mouse BM cells. In this experiment, BM cells were isolated from WT mice and then stimulated with IL-6 and GM-CSF. As shown in Figure 3A, PTP1B expression was significantly reduced in BM-derived MDSCs (BM-MDSCs) compared with BM cells, suggesting that PTP1B may negatively regulate the generation of MDSCs from BM leukocytes. Second, we compared the MDSC induction process from BM cells in PTP1B^−/−^ mice and WT littermates. In this experiment, BM cells isolated from WT and PTP1B^−/−^ mice were stimulated with IL-6 and GM-CSF and then assessed daily using antibodies against MDSC markers. The results clearly indicated that, under stimulation with IL-6 and GM-CSF, PTP1B^−/−^ BM cells produced MDSCs more rapidly than WT BM cells (Figure 3B).

Previous studies demonstrated that PTP1B inhibitor FRJ (Santa Cruz, CA, sc-222227) specifically inhibits PTP1B activity [25], whereas TNFα treatment increases the expression of PTP1B in adipocyte cell lines [26]. To further analyze the link between PTP1B and MDSCs, we treated BM cells from WT mice with PTP1B inhibitor FRJ or stimulator TNFα and then examined MDSC induction. Consistent with a role for PTP1B as a negative regulator during MDSC induction, treatment with the PTP1B inhibitor FRJ strongly promoted the induction of MDSCs from BM cells (Figure 3C). In contrast, TNFα treatment induced a dose-dependent increase in the expression level of PTP1B in BM cells (Figure 3D) and impaired the frequency of MDSCs derived from BM cells (Figure 3E).

### MDSCs Ameliorates Murine DSS-induced Colitis

**Figure 4:**
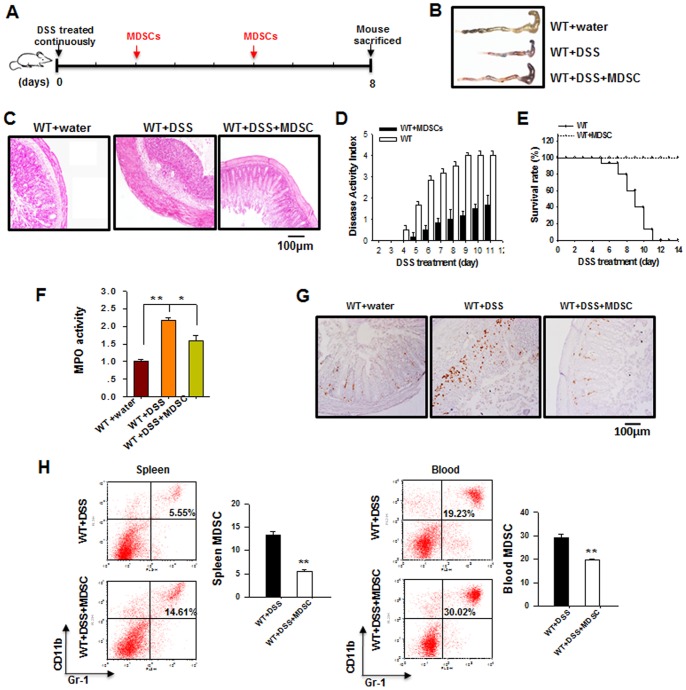
Adoptively transferred MDSCs exert a protective effect on DSS-induced mouse colitis. A) The experimental design. BM-induced MDSCs (5×10^6^ cells) were injected into mice through the tail vein. Note that WT mice that received adoptively transferred MDSCs have nearly normal length of the colon (B), almost intact colonic architecture (C), and lower DAI (D) but higher survival rates (E) than that in control mice without MDSC treatment, during DSS treatment. F–G) The relative activity of MPO (F) and anti-F4/80 staining in colons (G) from DSS-treated control mice and DSS-treated mice that received adoptively transferred MDSCs. H) The frequencies of MDSCs in the spleen and peripheral blood of DSS-treated control mice and DSS-treated mice that received adoptively transferred MDSCs. Data are representative of 3 replicated experiments with similar results (n = 5 per group per condition). Compared with WT-DSS, *, *p*<0.05, **, *p*<0.01.

Next, we characterized the role of MDSCs in murine DSS-induced experimental colitis. In this experiment, BM-MDSCs were adoptively transferred into WT mice via intravenous injection during the course of DSS treatment (Figure 4A). The adoptive transfer of BM-MDSCs clearly attenuated the colon tissue damage induced by DSS treatment, which is reflected by the nearly normal length of the colon (Figure 4B), intact colonic architecture (Figure 4C), and significant lower DAI (Figure 4D) but higher survival rate (Figure 4E) compared to mice without MDSC adoptive transfer. The adoptive transfer of BM-MDSCs into mice also strongly inhibited the infiltration of PMN (Figure 4F) and macrophages (Figure 4G) into colon. As expected, the adoptive transfer of BM-MDSCs markedly increased the frequency of MDSCs in mouse spleen and peripheral blood (Figure 4H). Interestingly, tracking the fluorescently labeled MDSCs in mice showed that an infiltration of the transferred MDSCs into the spleen sections of colitis mice but not the control mice (Figure S1), implicating that, under inflammation, adoptive transferred MDSCs migrated to mouse spleen where they could interact with T cells or other inflammatory leukocytes.

**Figure 5:**
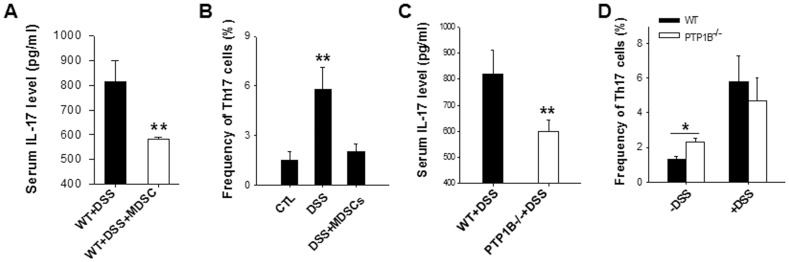
MDSCs decrease the frequency of Th17 cells in mice with DSS-induced colitis. WT mice were treated with 2.5% DSS to induce colitis, and during the same time, mice were injected with or without MDSCs induced from mouse BM cells. A) Adoptive transfer of MDSCs decreased serum IL-17 level in DSS-treated WT mice. B) The frequencies of Th17 cells in peripheral blood mononuclear cells (PBMCs) from WT mice that had or had not received BM-MDSCs. C) The level of serum IL-17 in PTP1B^−/−^ mice and WT mice. D) The frequencies of Th17 cells in PBMCs from PTP1B^−/−^ and WT mice with or without DSS treatment. Data are representative of 3 replicated experiments with similar results (n = 5 per group per condition). *, *p*<0.05, **, *p*<0.01.

Interleukin-17 (IL-17) is a major pro-inflammatory cytokine associated with colitis [27]–[30], and has been shown to be strikingly increased by DSS treatment [31]. As our data indicate that WT mice with adoptive transferring of MDSCs (Figure 5A) displayed a lower serum IL-17 level compared to WT mice in DSS-colitis, we hypothesize that MDSCs, which are expanded in PTP1B^−/−^ mice, may suppress the frequencies of IL-17–secreting Th17 cells. As shown in Figure 5B, during DSS treatment, the frequency of Th17 among peripheral blood mononuclear cells (PBMCs) decreased significantly in WT mice that had received BM-MDSCs compared to WT mice that had not received BM-MDSCs. In agreement with this, since PTP1B^−/−^ mice had a higher frequency of MDSCs than WT mice had, the level of serum IL-17 in PTP1B^−/−^ mice was significantly lower than that in WT mice (Figure 5C). However, to our surprise, we did not found the decrease of frequency of Th17 cells in PTP1B^−/−^ mice compared to WT mice after DSS treatment (Figure 5D).

### PTP1B Deficiency Promotes the Expansion of MDSCs via Increasing STAT3 Activity

**Figure 6:**
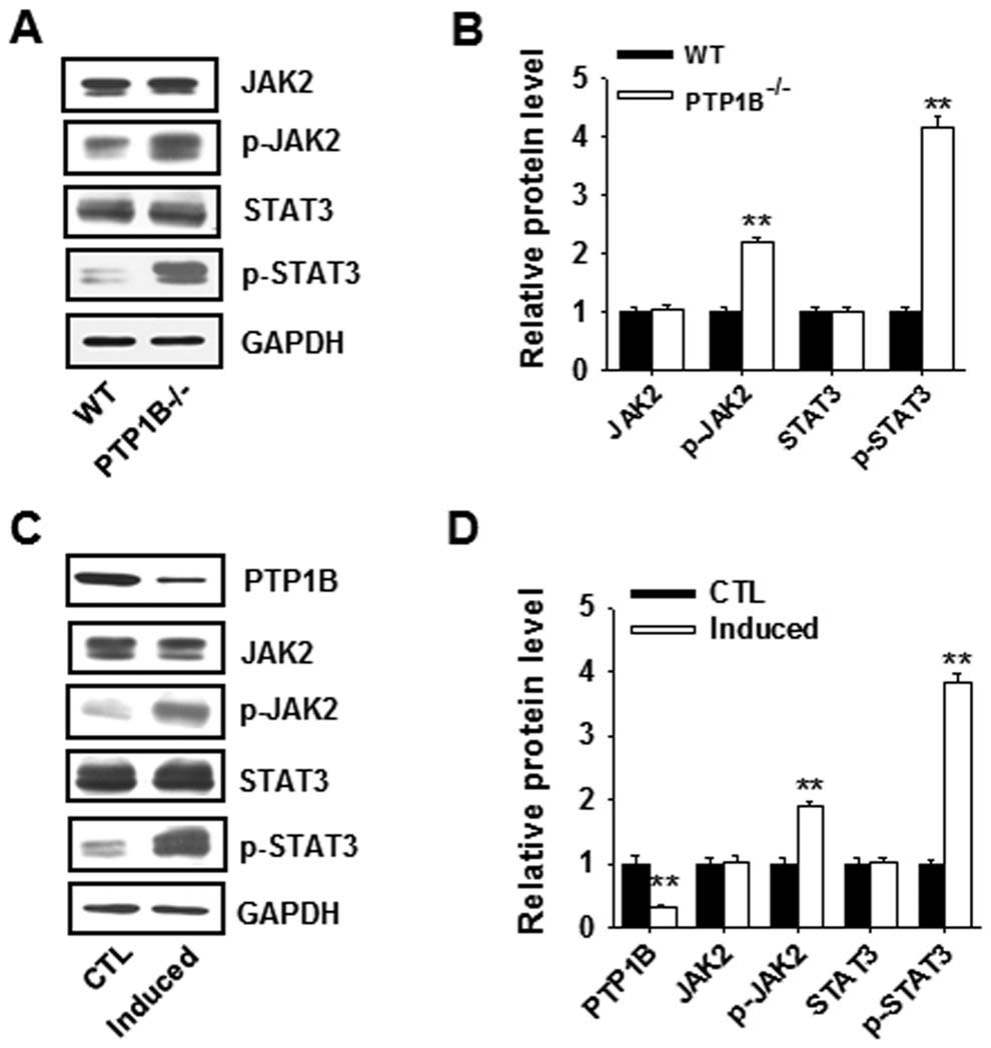
PTP1B deficiency promotes the induction of MDSCs from mouse BM cells via increasing JAK2/STAT3 activity. A–B) The protein expression levels of JAK2, p-JAK2, STAT3, p-STAT3 in BM cells from DSS-treated WT and PTP1B^−/−^ mice. C–D) The protein expression levels of PTP1B, JAK2, p-JAK2, STAT3, p-STAT3 in BM cells cultured in the presence or absence of cytokines (IL-6 and GM-CSF). Data are shown as the mean ± SD (n = 5). **, *p*<0.01.

Previous studies clearly indicated that the STAT3 signaling pathway is involved in the expansion of MDSCs [32]–[35]. Thus, we determined whether the expansion of MDSCs observed in PTP1B^−/−^ mice resulted from altered STAT3 activity. We first compared the levels of phosphorylated JAK2 (pJAK2) and STAT3 (pSTAT3), as well as total JAK2 and STAT3, in BM cells from WT and PTP1B^−/−^ mice. As shown in Figure 6, A and B, although BM cells isolated from WT and PTP1B^−/−^ mice have similar levels of total JAK2 and STAT3, the levels of pJAK2 and pSTAT3 are significantly higher in PTP1B^−/−^ BM cells than WT BM cells. The elevated levels of phosphorylated JAK2 and STAT3 in the BM cells of PTP1B^−/−^ mice are consistent with the reported ability of JAK2/STAT3 activity in promoting the expansion of MDSCs and the observed enhancement of MDSC expansion in PTP1B^−/−^ mice. The incubation of PTP1B^−/−^ BM cells with IL-6 and GM-CSF, two cytokines can induce BM cells into BM-MDSCs, greatly increased the levels of phosphorylated JAK2 and STAT3, confirming that pJAK2 and pSTAT3 are substrates of PTP1B (Figure 6, C and D).

## Discussion

Previous studies have demonstrated that PTP1B is not only an important regulator in insulin and leptin sensitivities, but also a critical factor in inflammatory regulation. The report by Zabolotny et al [26] clearly showed that PTP1B could be increased or activated by inflammatory factors such as TNFα. Recent studies have also identified the essential role of PTP1B in obesity-induced inflammation and peripheral insulin resistance during ageing [36] and regulating leukocyte recruitment during allergic inflammation [37]. In the present study, we demonstrated that PTP1B deficiency enhanced the activity of JAK2 and STAT3, thereby promoting the production of MDSCs, which, in turn, suppressed the inflammation and tissue damage induced by DSS treatment. As shown in Figure 1, PTP1B^−/−^ mice were considerably more resistant to DSS-induced colitis than WT mice. Compared with WT mice, PTP1B^−/−^ mice possessed more functional MDSCs in the BM, peripheral blood and spleen (Figure 2). An *in vitro* induction assay demonstrated that PTP1B is a negative regulator of MDSC production from BM cells (Figure 3A). Furthermore, kinetic studies indicated that the induction of MDSCs from BM cells was significantly more rapid in PTP1B^−/−^ mice than in WT mice (Figure 3B). More importantly, the causal relationship between PTP1B and MDSCs was investigated by altering PTP1B activity or expression using the PTP1B inhibitor FRJ or the PTP1B stimulator TNFα. The frequency of MDSCs was strongly increased by FRJ and effectively reduced by TNFα (Figure 3, C–E). The inhibitory effect of MDSCs on the induction of IBD by antigen-specific T cells was recently reported by Haile et al. [17]. In the present study, the protective role of MDSCs was confirmed in a murine DSS-induced colitis model by adoptively transferring BM-MDSCs into DSS-treated mice (Figure 4). It is worthy to mention that our result is different from that previously reported by Hassan et al [38]. Employing DSS-colitis model of heterozygous T cell protein tyrosine phosphatase (TC-PTP) mutant mice, the authors showed that heterozygous TC-PTP^+/−^ mice display increased susceptibility to systemic inflammation due to DSS-induced bowel epithelial damage. Compared to WT mice, heterozygous TC-PTP^+/−^ mice had a higher activity of Stat3, as well as Stat 1 and 5, and higher levels of pro-inflammatory cytokines under DSS treatment. Although the molecular basis underneath this difference remains unknown, these studies suggest that, TC-PTP and PTP1B, two phosphatases share 72% catalytic domain sequence identity, may act differently and nonredundantly in cellular signaling.

IL-17 is a major proinflammatory cytokine associated with colitis. IL-17-producing cells, mainly T helper (Th)17 cells, have been reported to play critical roles in various immune-mediated diseases, including inflammatory bowel diseases (IBD) [39]. In a separate study, we had reported that mouse plasma IL-17 was strikingly increased by DSS treatment [31]. Here we found that PTP1B^−/−^ mice contained a lower serum IL-17 level compared to WT mice in DSS-colitis, suggesting that IL-17-producing cells are likely suppressed in PTP1B^−/−^ mice under inflammatory condition. As shown in Figure 5, the frequency of Th17 in peripheral blood mononuclear cells decreased significantly in DSS-treated WT mice after receiving BM-MDSCs, implying that MDSCs may protect mice from DSS-colitis via suppressing the frequencies of IL-17-producing Th17 cells. Of course, the present study only tested the level of IL-17 in serum and the frequency of Th17 cells in peripheral blood. It remains unknown whether the IL-17 level and Th17 cell population in intestinal and colon tissues were decreased by infiltrated MDSCs. Future study is required to clarify this critical issue.

A recent study indicated that JAK2 is a substrate of PTP1B [40] and pointed to an essential role for PTP1B in modulating JAK2/STAT3 signaling. This negative regulatory role of PTP1B in JAK/STAT-mediated signaling was demonstrated in two strains of PTP1B^−/−^ mice [41], [42] and by the induction of various cytokines, such as leptin [43] and prolactin [44]. In the present study, we clearly demonstrate that JAK2 and STAT3 are substrates of PTP1B and that PTP1B deficiency significantly enhances the phosphorylation of JAK2 and STAT3 (Figure 6). Through the modulation of MDSCs, PTP1B plays a central role in regulating intestinal inflammation. Intestinal inflammation, likely via IL-17, TNFα or other inflammatory factors, increases the expression of PTP1B, which then suppresses the activities of JAK2/STAT3, decreases the production of MDSCs and subsequently exacerbates intestinal inflammation. Thus, the inflammation-PTP1B-MDSC cycle functions as a positive-feedback loop in the dynamic process of chronic inflammation. Furthermore, the essential role of PTP1B in regulating inflammation provides a novel therapeutic target for controlling inflammation.

In summary, our study provides the first evidences that PTP1B dephosphorylates STAT3 and functions as a critical negative regulator of the generation of MDSCs. Therefore, PTP1B deficiency promotes the expansion of MDSCs, which in turn, decreases the infiltration of neutrophils, reduces the level of serum IL-17 and ameliorates murine experimental colitis.

## Supporting Information

Figure S1 **Infiltration of fluorescently labeled MDSCs (green) into the spleen sections of DSS colitis mice but not control mice.**
(DOCX)
