# Supplementary material for: Protein Tyrosine Phosphatase 1B Deficiency Ameliorates Murine Experimental Colitis via the Expansion of Myeloid-Derived Suppressor Cells
Source: PLoS One. 2013 Aug 9;8(8):e70828. doi: 10.1371/journal.pone.0070828 (PMC3739765; doi:10.1371/journal.pone.0070828)
Supplement: Figure S1 — Infiltration of fluorescently labeled MDSCs (green) into the spleen sections of DSS colitis mice but not control mice. (DOCX) [file pone.0070828.s001.docx]

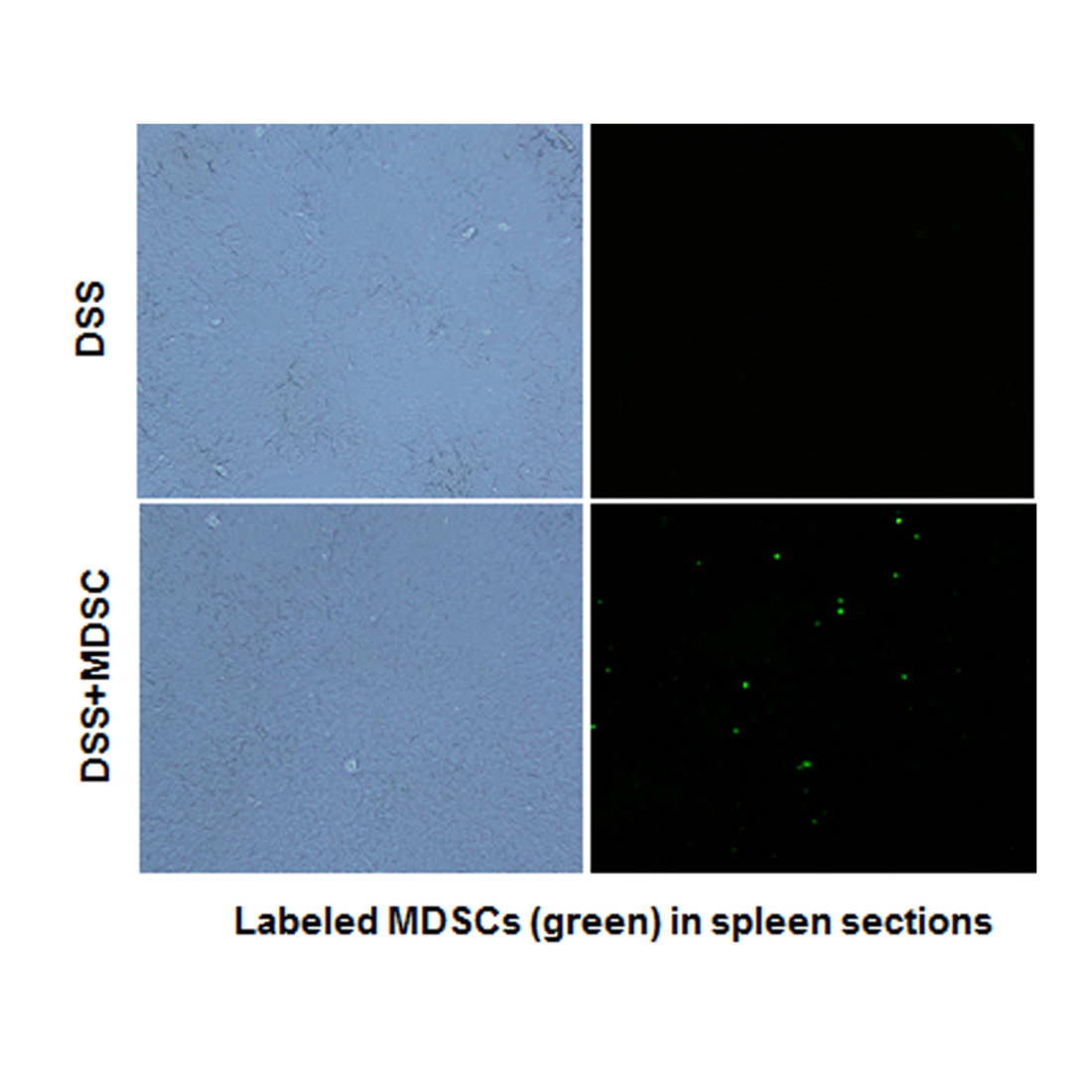


Figure S1. Infiltration of fluorescently labeled MDSCs (green) into the spleen sections of DSS colitis mice but not control mice.
